# Supplementary material for: Molecular consequences of acute versus chronic CDK12 loss in prostate carcinoma nominates distinct therapeutic strategies
Source: bioRxiv. 2025 May 19:2024.07.16.603734. Originally published 2024 Jul 19. Preprint. [Version 2] doi: 10.1101/2024.07.16.603734 (PMC11275783; doi:10.1101/2024.07.16.603734)
Supplement: Supplement 5 [file NIHPP2024.07.16.603734v2-supplement-5.pdf]

bioRxiv preprint doi: https://doi.org/10.1101/2024.07.16.603734; this version posted May 19, 2025. The copyright holder for this preprint (which was not certified by peer review) is the author/funder, who has granted bioRxiv a license to display the preprint in perpetuity. It is made available under aCC-BY 4.0 International license.

Supplemental Figure S1. CDK12 genomic alterations and tandem duplication phenotypes

(a) Tandem duplication (TD) size analysis confirms multiple modes of TD lengths (top) in whole exome (WES; orange) and whole genome sequencing (WGS; blue) data for 46 tumors with a TDP. Fitting mixtures of Gaussian distributions to each of 26 WES and 20 WGS samples provided estimates of Gaussian means. The distribution of these mean values across 26 WES cases and 20 WGS cases are shown in red dotted lines; samples may have multiple mean values, one for each estimated mixture. The mixture means are categorized into six possible TD Groups based on the length ranges (bottom). The proportion of each TD Group are shown for the four CRPC cohorts.

(b) The proportions of *CDK12*, *BRCA2*, and *TP53* genomic alteration status, including mono-allelic and bi-allelic losses and intact, for the 46 TDP cases compared across TD Groups.

Supplemental Figure S2. Gene expression alterations in *CDK12^BAL^* prostate cancers.

(a) GSEA of CDK12-mut up and down signatures reported in Wu et al., 2018 applied to mCRPC cohorts with versus without *CDK12^BAL^*. (Normalized enrichment scores (NES) shown in heatmap on plot, all enrichment false discovery rates (FDR) <0.0001).

(b-c) Overlap of differentially expressed genes up-regulated (**c**) or down-regulated **(d)** across mCRPC cohorts with versus without *CDK12^BAL^* (significance level shown on plot.)

(d-g) RNAseq based quantitation of AR pathway activity in mCRPC cohorts with versus without *CDK12^BAL^* (GSVA scores; Wilcoxon rank test p-values shown).

(h) Differential expression of genes contained within *CDK12^BAL^*-specific and recurrent fusions. * = Up-regulated or ^ down-regulated with p<0.05, FC>abs(2) in at least 1 cohort.

(i-l) RNAseq based transcript abundance levels of ATM in mCRPC cohorts with versus without *CDK12^BAL^* (Wilcoxon rank test p-values shown).

Supplemental Figure S3. APA usage in *CDK12^BAL^* prostate cancers.

(a-c) Association of increased/unchanged or decreased (p<0.05; FC<-2) transcript abundance levels in relation to gene size features in tumors without or with CDK12^{BAL} in the HMF, SU2C-WC and UW mCRPC cohorts.

(d-f) APAlyzer analysis for up- and down-regulated APA usage using RNA-seq from mCRPC data sets comparing CDK12^{BAL} cases vs CDK12(intact) controls: (c) TCGA-PRAD, (d) SU2C-I, and (e) HMF. RED = relative expression difference; each point is a different APA site.

bioRxiv preprint doi: https://doi.org/10.1101/2024.07.16.603734; this version posted May 19, 2025. The copyright holder for this preprint (which was not certified by peer review) is the author/funder, who has granted bioRxiv a license to display the preprint in perpetuity. It is made available under aCC-BY 4.0 International license.

(g) Transcript pile-up of reads mapping to exons demonstrating increased transcript reads corresponding to an IPA in the ATM gene in TCGA prostate (PRAD) and ovarian primary tumors with CDK12 alterations versus tumors with intact CDK12 and diminished transcripts mapping to the distal 3' exon.

Supplemental Figure S4. Effects of acute CDK12 inhibition.

(a) Treatment with ActD, Palbo, and SR4835 all lead to downregulation of longer transcripts. Distribution by transcript length (nucleotides/nt) of downregulated genes by RNA-seq (<-2 fold, FDR <0.05, 'n' depicted on plot) in LNCaP and LuCaP35_CL prostate cancer cell lines following six hours of exposure to vehicle (DMSO), CDK4/6 inhibitor palbociclib (Palbo, 10uM), broad RNA Pol-II inhibitor actinomycin D (ActD, 5ug/mL), or CDK12/13 inhibitor SR4835 (200nM) (n=3). Plots were made with ShinyGO 0.80 (76) and show significance (t-test) of downregulated vs upregulated or unchanged genes.

(b) CDK12 inhibition increases the number and ratio of upregulated APAs**.** Table with the number of APA sites down (DN), no change (NC), or up (UP) upon treatment vs vehicle and the ratio UP/DOWN, indicating any skew towards the IPA phenotype.

(c-d) Cell arrest and CDK12 inhibition both lead to HR pathway downregulation. **(c)** Selected KEGG pathway enrichment for DNA-repair related pathways focusing on changes from general G1/S arrest (palbociclib) vs CDK12 inhibition (SR4835). (d) Many SR4835 downregulated pathways overlap with cell cycle arrest. Venn diagram showing high overlap of KEGG pathways downregulated (NES <-1) with acute palbociclib or SR4835 treatment (6h).

(e-f) Effects of pharmacological CDK12/13 inhibition. Similar experiments as in Fig. 4f using LNCaP (e) and Skov3 (f) cells showing the effect on DNA repair and apoptotic proteins with SR4835 treatment with or without Z-VAD caspase inhibitor.

(g) LNCaP and C42B were transduced with lentivirus containing dual sgRNAs against CDK12 or non-targeting controls. Lysates were harvested 7 days post infection and analyzed by immunoblot.

(h) LuCaP189.4 carries bi-allelic loss of function *CDK12* mutations. Diagram showing the two frameshift mutations carried by the LuCaP189.4 PDX upstream of the key functional kinase domain (yellow).

(i) LuCaP 189.4 does not express CDK12 protein. Immunohistochemical (IHC) staining (with amplification) for CDK12 on FFPE sections from LuCaP PDX tumors or cell spots from LuCaP189.4_CL and LuCaP189.4-CDK12 cell lines. Counterstained with hematoxylin.

(j) LuCaP189.4 shows hallmark TDP genomic pattern. Copy number plot from exome-seq of three LuCaP PDX lines (78CR, 174.1, and 189.4).

(k) *CDK12* knockout lines grow slower than parental lines. GFP tagged cells were grown for seven days and monitored by GFP imaging. Graphs show GFP confluence normalized to day 1 for each line with

bioRxiv preprint doi: https://doi.org/10.1101/2024.07.16.603734; this version posted May 19, 2025. The copyright holder for this preprint (which was not certified by peer review) is the author/funder, who has granted bioRxiv a license to display the preprint in perpetuity. It is made available under aCC-BY 4.0 International license.

mean-/+stdev (n=5). Significance vs parental line was determined by two way ANOVA.

Supplemental Figure S5. Validation of 22Rv1 CDK12 KO lines.

(a) PCR was performed on genomic DNA from 22Rv1 lines to amplify the sgRNA targeted sites in *CDK12* exon1 and exon4. Note the large products in KO2 (exon1) and KO5 (exon 4) indicating large genomic insertion events. **(b)** RNA-seq reads from the 22Rv1 lines show presence of frameshift indels in the CRISPR clones. **(c)** Low coverage WGS was performed on the clones and plotted for copy number alterations, with no obvious sign of a TDP pattern (as can be seen in Fig. 2a and S4g).

Supplemental Figure S6. Assessments of HR competency in cells with CDK12 loss.

(a) Stable CDK12(-) cells show fewer upregulated intronic APAs. Table with the number of APA sites down (DN), no change (NC), or up (UP) in isogenic paired models (CRISPR KO clones vs parental, or 189.4-CDK12 vs 189.4-vec). The ratio UP/DOWN indicates skew towards the IPA phenotype.

(b) Validation of Tet-shRNA lines. Western blot with lysates from Tet-shRNA lines treated four days -/+ 100ng/mL doxycycline.

(c) LuCaP189.4_CL cells are RAD51 competent. Irradiation and immunostaining (same as in Fig 5f). Cells were exposed to 6Gy IR and fixed at 3h. Immunofluorescence staining was performed for γH2A.X and RAD51 and images were acquired by confocal microscopy. Left: representative images (white: DAPI, green: γH2A.X, purple: RAD51). Right: quantification of images (~200-500 cells analyzed per treatment). Line is at mean and significance was determined by unpaired t-test (Mann-Whitney).

(d-e) *CDK12* knockdown does not prevent RAD51 foci. Additional immunostaining (same as in Fig 5f) using LNCaP (d) and Skov3 (e) with Tet-shCDK12 or Tet-shBRCA2. Cells were treated four days -/+ dox. Graphs show mean with significance determined by one-way ANOVA (Kruskal-Wallis).

Supplemental Figure S7. Drug sensitivities in prostate cancers with CDK12 loss.

(a-b) CDK12 loss does not confer HRd-expected platinum or PARPi sensitivity. Dose response curves for prostate cancer (a) or ovarian cancer (b) cell lines treated 8 days with carboplatin (n=3 for prostate lines, n=4 for ovarian lines). EC50 values are shown on the legend.

(c) Ovarian cancer cells (n=4) were treated 12 days with olaparib.

(d) Prostate cancer lines and UWB1.289 (n=3) were treated 12 days with olaparib.

(e) Prostate cancer lines and UWB1.289 (n=3) were treated 8 days with rucaparib.

bioRxiv preprint doi: https://doi.org/10.1101/2024.07.16.603734; this version posted May 19, 2025. The copyright holder for this preprint (which was not certified by peer review) is the author/funder, who has granted bioRxiv a license to display the preprint in perpetuity. It is made available under aCC-BY 4.0 International license.

(f-g) LuCaP189.4 organoids do not show obvious PARPi sensitivity. LuCaP PDX tumors were dissociated into organoids and treated (at passage 3) with olaparib (f) or rucaparib (g) for 14 days. Plots show mean-/+stdev (n=4).

(h-j) CDK13 sgRNA is detrimental in 22Rv1 lacking *CDK12*. GFP tagged 22Rv1 or 22Rv1-CDK12-KO5 were transduced with sgRNAs and monitored by imaging. Example images are in (h). Plots show growth rates for 22Rv1 (i) and 22Rv1-CDK12-KO5 (j) by confluence (%GFP-/+stdev, n=5) with significance vs sgAAVS1 determined by two-way ANOVA.

(k-n) LuCaP189.4_CL shows sensitivity to CDK13 inhibition. Dose response curves from four prostate cancer lines, including CDK12^{BAL} LuCaP189.4_CL, treated six days with the CDK12/13 inhibitors SR4835 (k) or THZ531 (l). 3D cultured spheroids were treated three days with THZ531, confirming LuCaP189.4 heightened sensitivity (m). Calculated EC50 values (nM) are listed in table (n).

(o) APAlyzer analysis from SR4835 treated PDX tumors. Three tumors of each group from Fig. 6j plus three day treated LuCaP189.4 were analyzed by RNA-seq. The table shows the number of intronic APA sites down (DN), no change (NC), or up (UP) in treated vs vehicle comparisons. The ratio UP/DOWN indicates skew towards the IPA phenotype.

Supplemental Figure S8. Effects of WEE1, ATR and CHEK1 inhibitors toward prostate cancers with CDK12 loss.

(a-c) One CDK12-KO prostate line, 22Rv1-CDK12-KO5, shows increased sensitivity to WEE1 inhibition. Prostate cancer lines were treated with WEE1 inhibitors adavosertib/MK-1775 (a) and PD0166285 (b) for four days. Dose response curves show relative viability vs drug concentration. Plots show mean-/+stdev (n=4). Legend and EC50 values (µM) are shown in (c).

(d-f) CDK12-KO prostate lines do not show sensitivity to ATR inhibitors. Prostate cancer lines were treated with ATR inhibitors berzosertib/VX-970 (d) and elimusertib/BAY-1895344 (e) for four days. Dose response curves show relative viability vs drug concentration. Plots show mean-/+stdev (n=4). Legend and EC50 values (nM) are shown in (f).

(g-i) One CDK12-KO prostate line, 22Rv1-CDK12-KO5, shows increased sensitivity to CHEK1 inhibition. Prostate cancer lines were treated with CHEK1 inhibitors rabusertib (g) and MK-8776 (h) for four days. Dose response curves show relative viability vs drug concentration. Plots show mean-/+stdev (n=4). Legend and EC50 values (µM) are shown in (i).

FIGURE S1

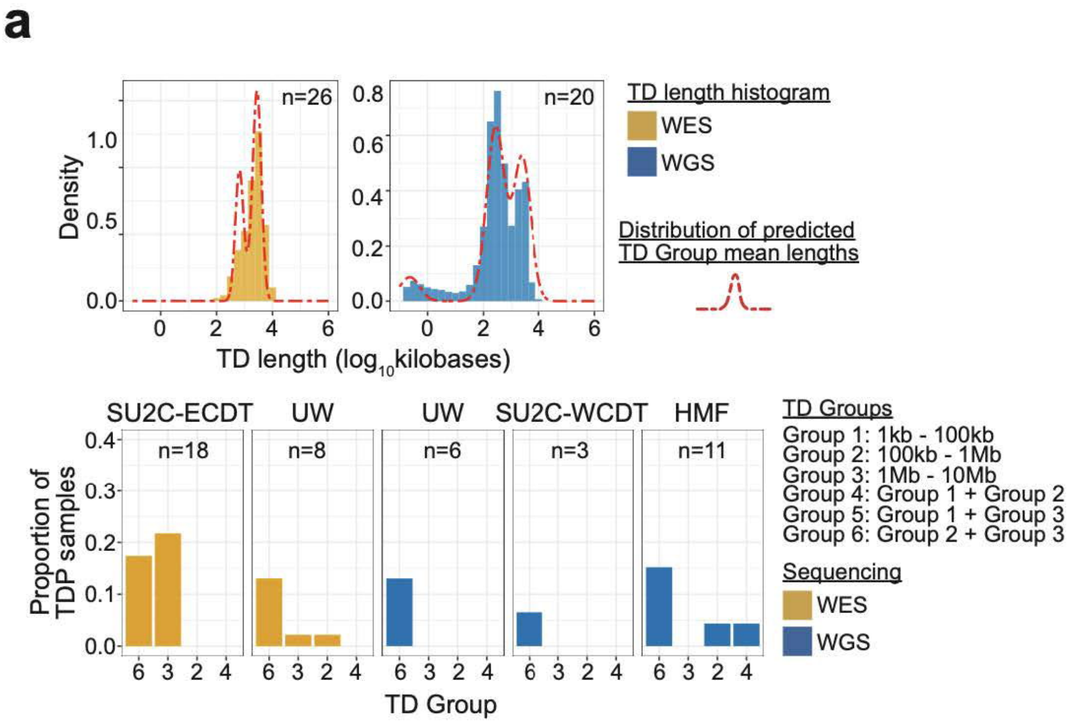

a
n=26
n=20
Density
TD length (log10kilobases)
TD length histogram
WES
WGS
Distribution of predicted
TD Group mean lengths
SU2C-ECDT
UW
UW
SU2C-WCDT
HMF
n=18
n=8
n=6
n=3
n=11
Proportion of
TDP samples
TD Group
TD Groups
Group 1: 1kb - 100kb
Group 2: 100kb - 1Mb
Group 3: 1Mb - 10Mb
Group 4: Group 1 + Group 2
Group 5: Group 1 + Group 3
Group 6: Group 2 + Group 3
Sequencing
WES
WGS

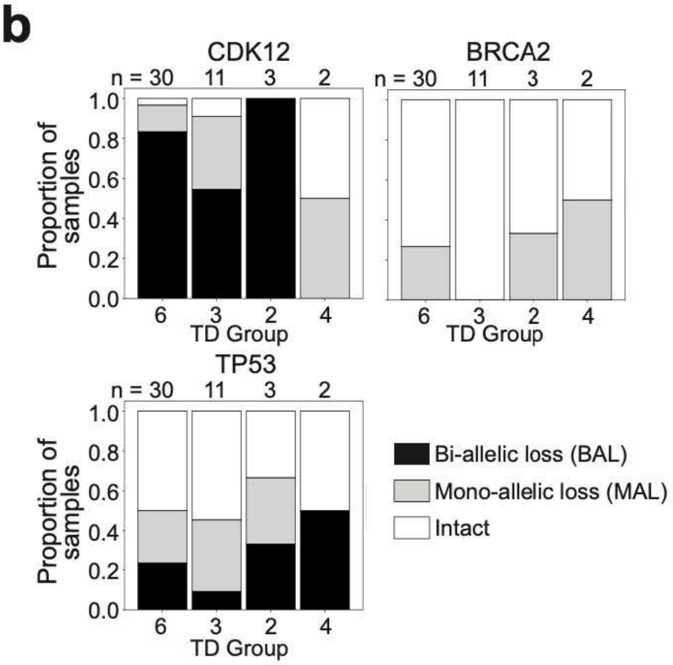

b
CDK12
BRCA2
TP53
n = 30
11
3
2
Proportion of
samples
TD Group
Bi-allelic loss (BAL)
Mono-allelic loss (MAL)
Intact

FIGURE S2

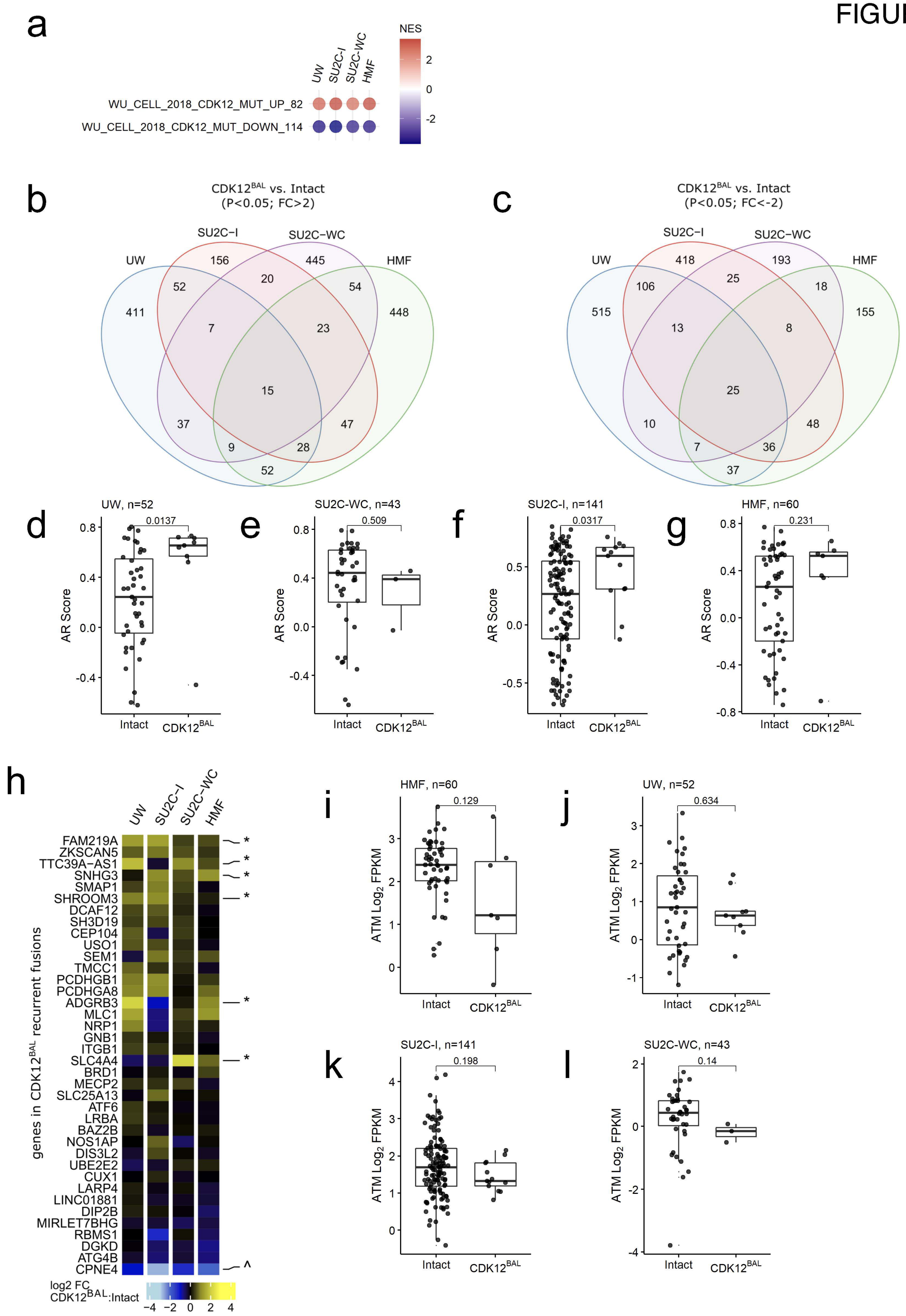

a
NES
UW
SU2C-I
SU2C-WC
HMF
WU_CELL_2018_CDK12_MUT_UP_82
WU_CELL_2018_CDK12_MUT_DOWN_114
2
0
-2
b
CDK12BAL vs. Intact
(P<0.05; FC>2)
UW
SU2C-I
SU2C-WC
HMF
411 156 445 448 52 20 54 7 23 15 37 47 9 28 52
c
CDK12BAL vs. Intact
(P<0.05; FC<-2)
UW
SU2C-I
SU2C-WC
HMF
515 418 193 155 106 25 18 13 8 25 10 48 7 36 37
d
UW, n=52
0.0137
AR Score
Intact
CDK12BAL
e
SU2C-WC, n=43
0.509
AR Score
Intact
CDK12BAL
f
SU2C-I, n=141
0.0317
AR Score
Intact
CDK12BAL
g
HMF, n=60
0.231
AR Score
Intact
CDK12BAL
h
UW
SU2C-I
SU2C-WC
HMF
genes in CDK12BAL recurrent fusions
FAM219A
ZKSCAN5
TTC39A-AS1
SNHG3
SMAP1
SHROOM3
DCAF12
SH3D19
CEP104
USO1
SEM1
TMCC1
PCDHGB1
PCDHGA8
ADGRB3
MLC1
NRP1
GNB1
ITGB1
SLC4A4
BRD1
MECP2
SLC25A13
ATF6
LRBA
BAZ2B
NOS1AP
DIS3L2
UBE2E2
CUX1
LARP4
LINC01881
DIP2B
MIRLET7BHG
RBMS1
DGKD
ATG4B
CPNE4
log2 FC
CDK12BAL:Intact
−4 −2 0 2 4
i
HMF, n=60
0.129
ATM Log2 FPKM
Intact
CDK12BAL
j
UW, n=52
0.634
ATM Log2 FPKM
Intact
CDK12BAL
k
SU2C-I, n=141
0.198
ATM Log2 FPKM
Intact
CDK12BAL
l
SU2C-WC, n=43
0.14
ATM Log2 FPKM
Intact
CDK12BAL

FIGURE S3

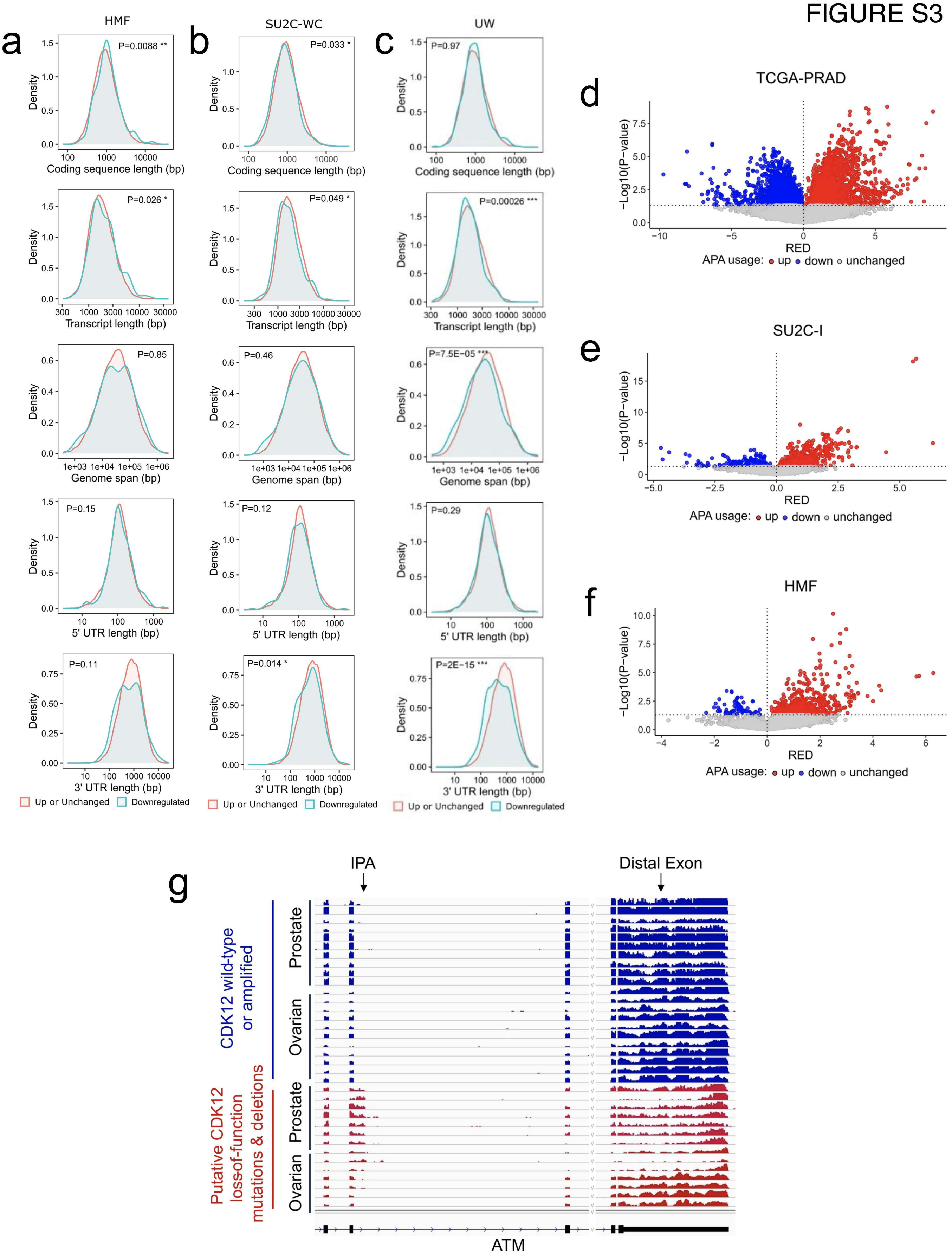

a
HMF
P=0.0088 **
P=0.026 *
P=0.85
P=0.15
P=0.11
b
SU2C-WC
P=0.033 *
P=0.049 *
P=0.46
P=0.12
P=0.014 *
c
UW
P=0.97
P=0.00026 ***
P=7.5E−05 ***
P=0.29
P=2E−15 ***
Density
Coding sequence length (bp)
Transcript length (bp)
Genome span (bp)
5' UTR length (bp)
3' UTR length (bp)
Up or Unchanged
Downregulated
d
TCGA-PRAD
e
SU2C-I
f
HMF
−Log10(P−value)
RED
APA usage: up down unchanged
g
IPA
Distal Exon
CDK12 wild-type or amplified
Putative CDK12 loss-of-function mutations & deletions
Prostate
Ovarian
ATM

FIGURE S4

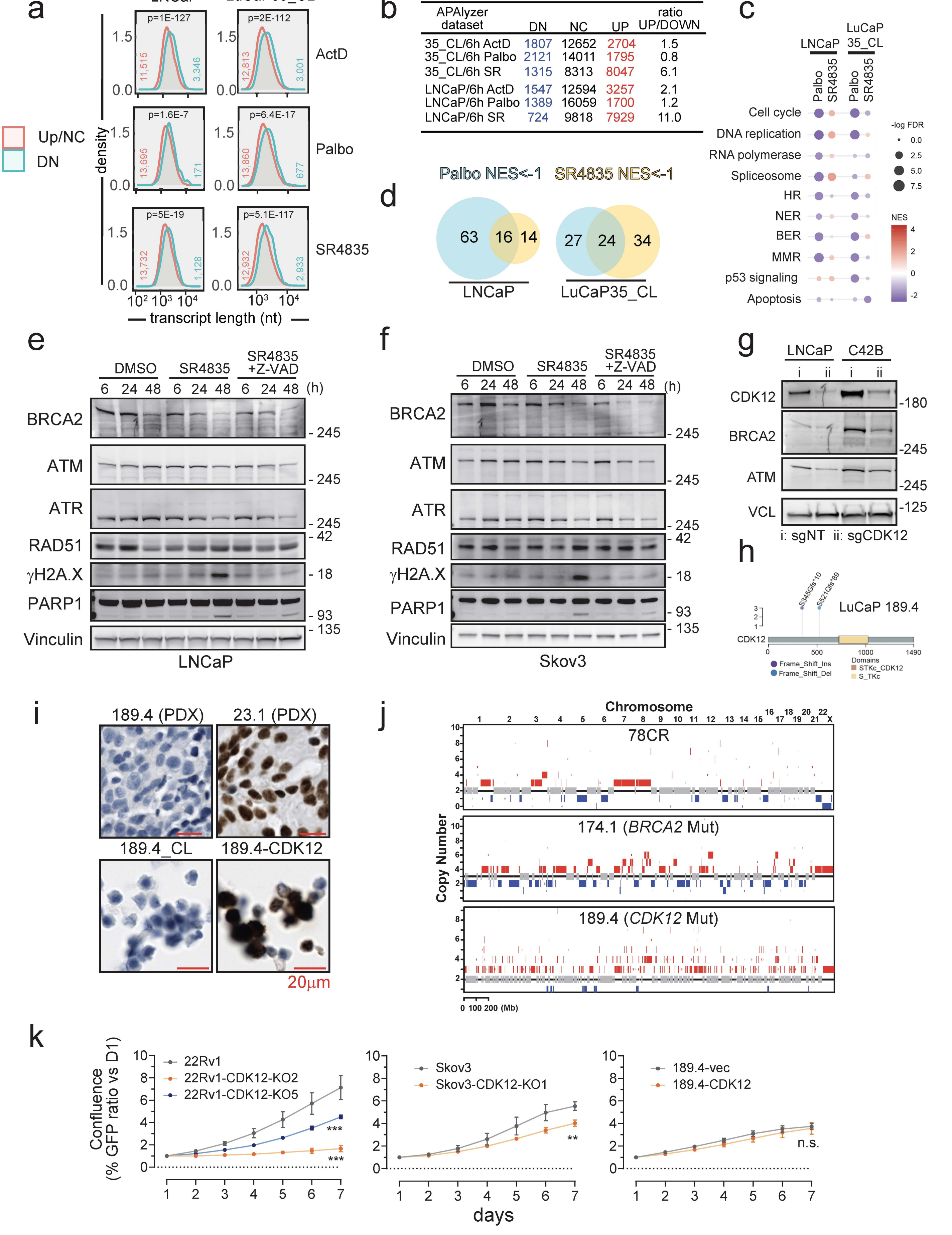
a
LNCaP
LuCaP35_CL
p=1E-127
p=2E-112
ActD
p=1.6E-7
p=6.4E-17
Palbo
p=5E-19
p=5.1E-117
SR4835
Up/NC
DN
density
transcript length (nt)
b
APAlyzer dataset | DN | NC | UP | ratio UP/DOWN
35_CL/6h ActD | 1807 | 12652 | 2704 | 1.5
35_CL/6h Palbo | 2121 | 14011 | 1795 | 0.8
35_CL/6h SR | 1315 | 8313 | 8047 | 6.1
LNCaP/6h ActD | 1547 | 12594 | 3257 | 2.1
LNCaP/6h Palbo | 1389 | 16059 | 1700 | 1.2
LNCaP/6h SR | 724 | 9818 | 7929 | 11.0
c
LNCaP
LuCaP 35_CL
Palbo
SR4835
Cell cycle
DNA replication
RNA polymerase
Spliceosome
HR
NER
BER
MMR
p53 signaling
Apoptosis
-log FDR
NES
d
Palbo NES<-1
SR4835 NES<-1
63 16 14
27 24 34
LNCaP
LuCaP35_CL
e
DMSO
SR4835
SR4835 +Z-VAD
6 24 48 (h)
BRCA2
ATM
ATR
RAD51
γH2A.X
PARP1
Vinculin
LNCaP
f
Skov3
g
LNCaP
C42B
CDK12
BRCA2
ATM
VCL
i: sgNT ii: sgCDK12
h
S345Gfs*10
S521Qfs*89
LuCaP 189.4
CDK12
Frame_Shift_Ins
Frame_Shift_Del
Domains
STKc_CDK12
S_TKc
i
189.4 (PDX)
23.1 (PDX)
189.4_CL
189.4-CDK12
20μm
j
Chromosome
78CR
174.1 (BRCA2 Mut)
189.4 (CDK12 Mut)
Copy Number
0 100 200 (Mb)
k
Confluence (% GFP ratio vs D1)
22Rv1
22Rv1-CDK12-KO2
22Rv1-CDK12-KO5
Skov3
Skov3-CDK12-KO1
189.4-vec
189.4-CDK12
***
**
n.s.
days

FIGURE S5

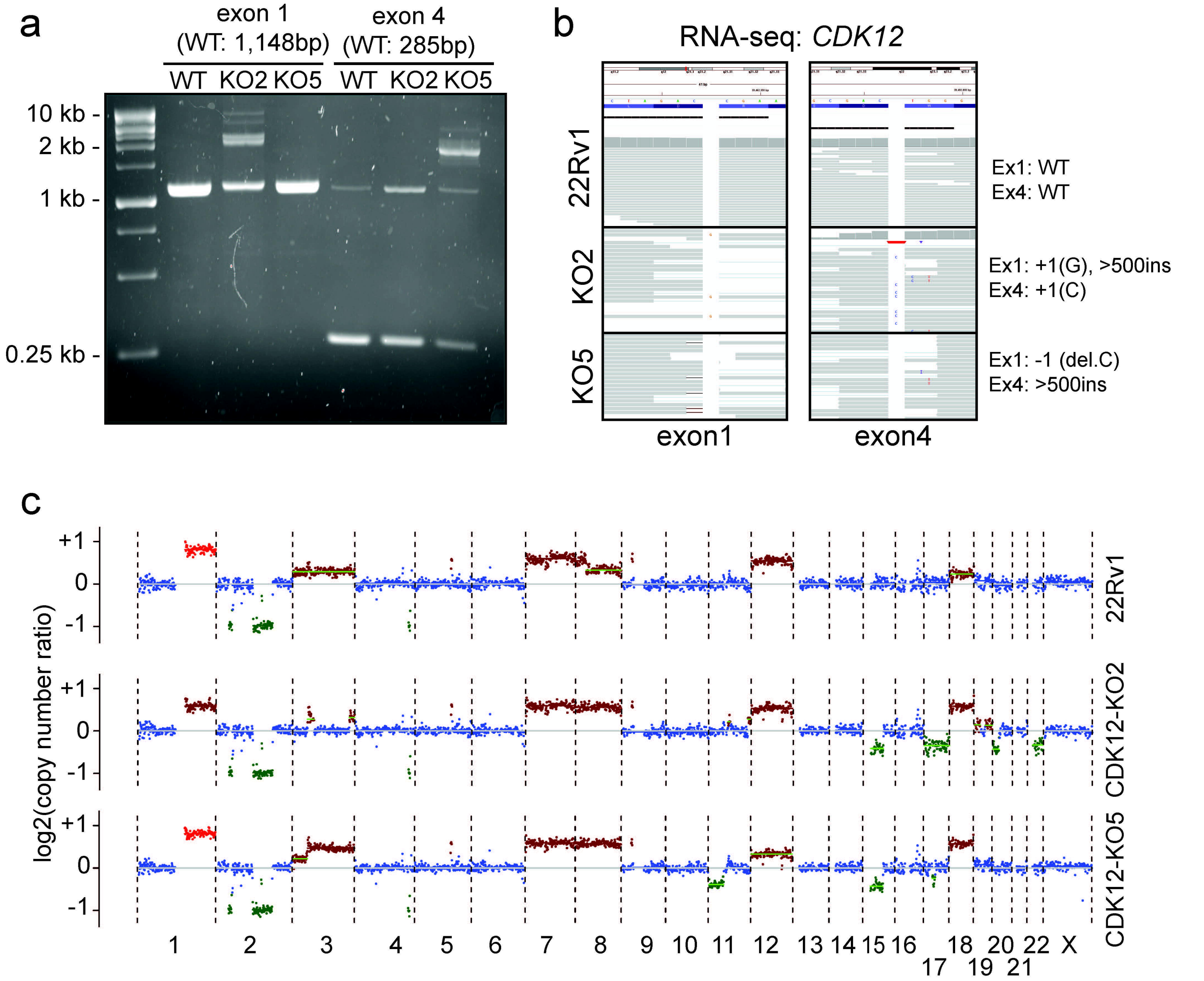

a
exon 1
(WT: 1,148bp)
exon 4
(WT: 285bp)
WT KO2 KO5 WT KO2 KO5
10 kb
2 kb
1 kb
0.25 kb
b
RNA-seq: CDK12
22Rv1
KO2
KO5
exon1
exon4
Ex1: WT
Ex4: WT
Ex1: +1(G), >500ins
Ex4: +1(C)
Ex1: -1 (del.C)
Ex4: >500ins
c
log2(copy number ratio)
+1
0
-1
22Rv1
CDK12-KO2
CDK12-KO5
1 2 3 4 5 6 7 8 9 10 11 12 13 14 15 16 17 18 19 20 21 22 X

FIGURE S6

a

APAlyzer dataset	DN	NC	UP	ratio UP/DOWN
22Rv1-KO2	2651	14322	2161	0.8
22Rv1-KO5	1926	14561	2350	1.2
Skov3-KO1	1229	12259	3347	2.7
189.4-CDK12	1791	16031	804	0.4

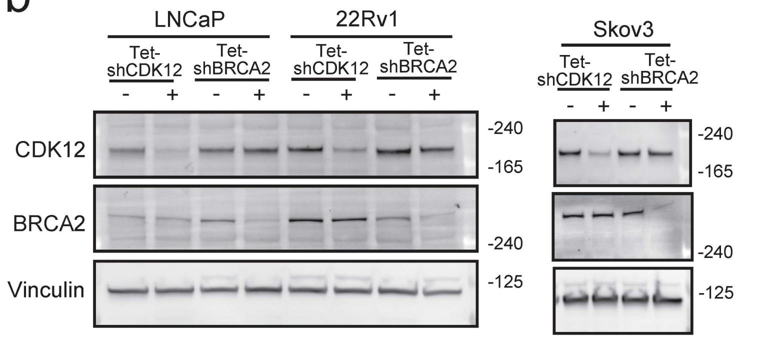

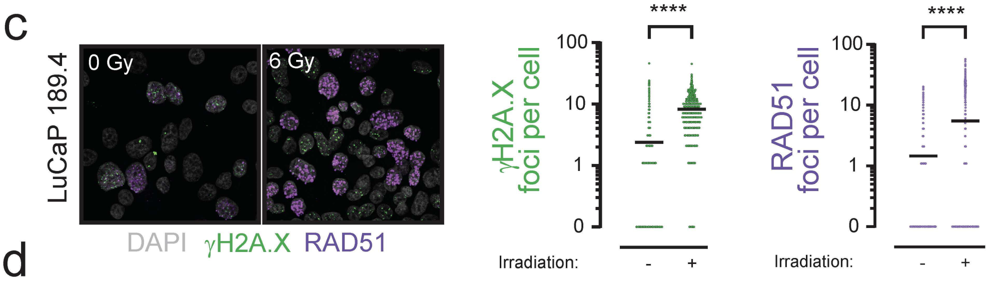

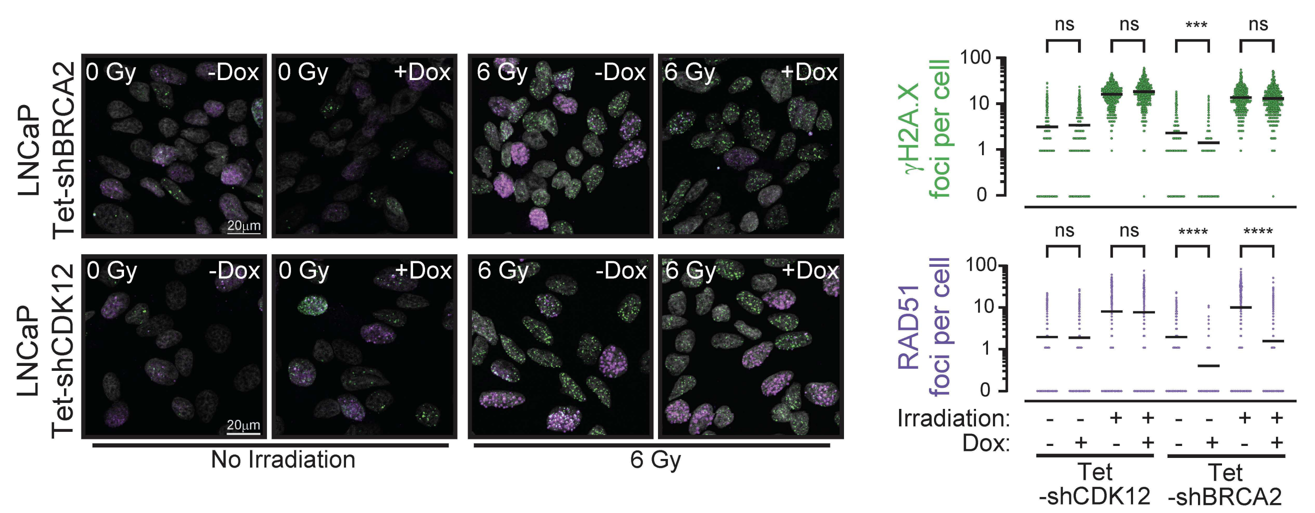

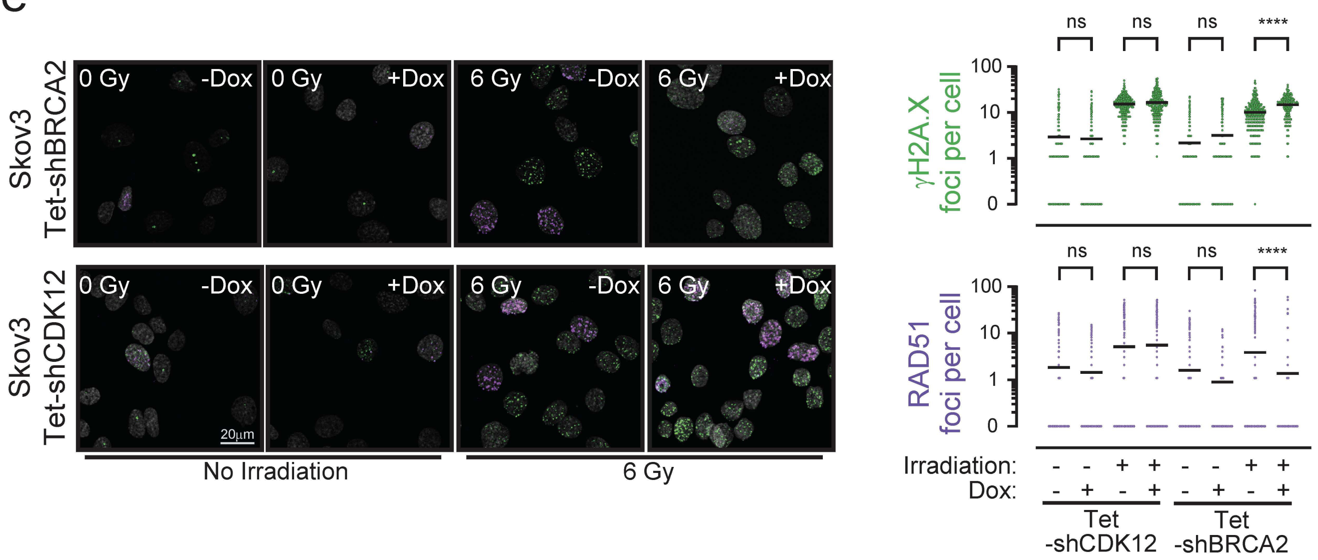

FIGURE S7

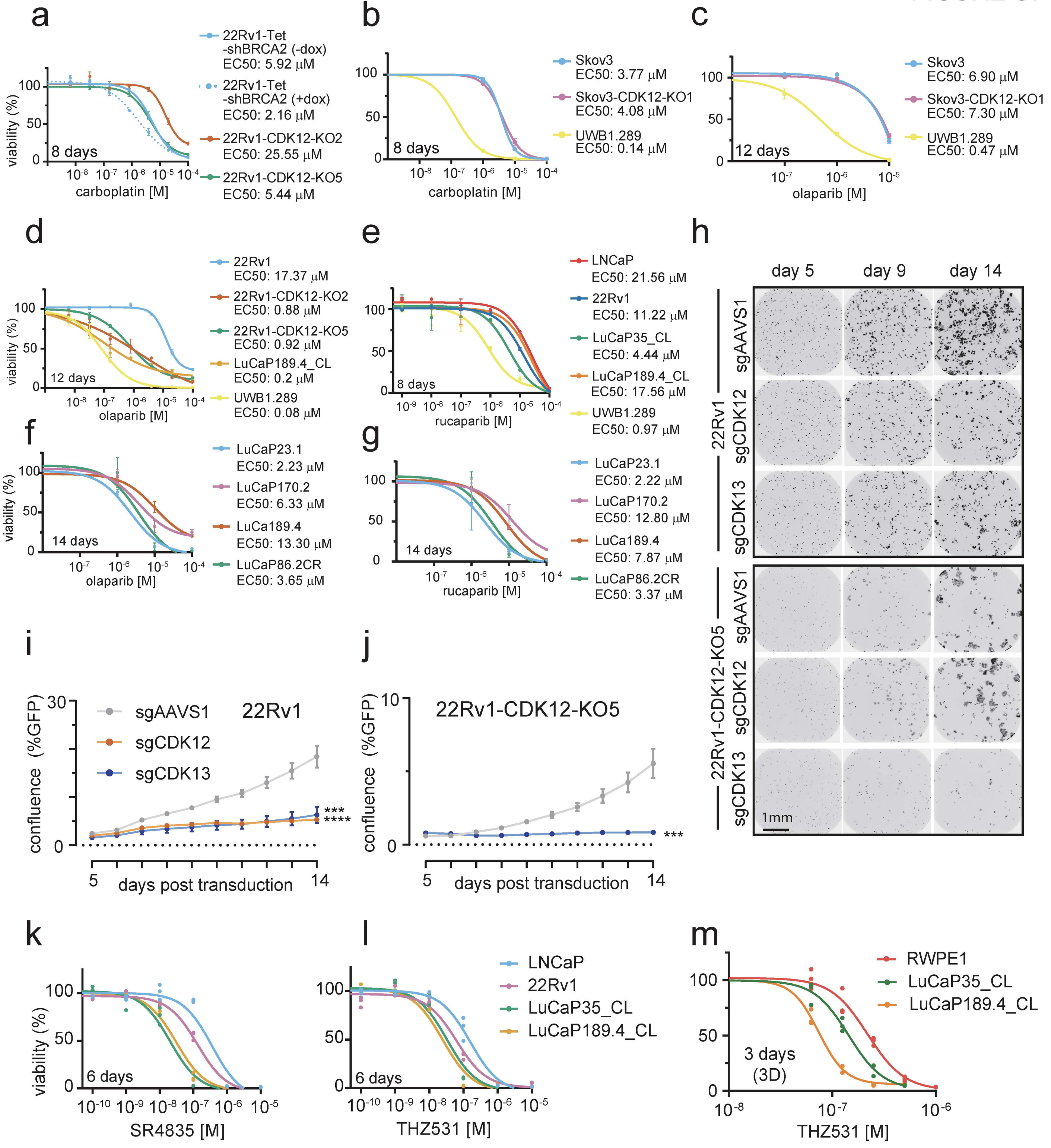

n

EC50 [nM]	SR4835 6d, 2D	THZ531 6d, 2D	THZ531 3d, 3D
LNCaP	297	145	
22Rv1	114	60	
LuCaP35_CL	20	33	145
LuCaP189.4_CL	29	25	77
RWPE1			223

o

APAlyzer dataset	DN	NC	UP	ratio UP/DOWN
136/28d	242	20275	157	0.6
35/28d	345	16336	342	1.0
189.4/28d	324	18165	339	1.0
189.4/3d	763	13096	2958	3.9

FIGURE S8

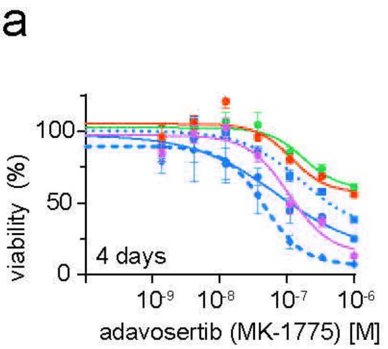

c

EC50 [μM]	WEE1 inhibitors adavosertib	PD0166285
LNCaP	0.13	0.06
189.4-vec	>1	0.41
189.4-CDK12	>1	0.53
22Rv1	0.11	0.37
22Rv1-KO2	0.43	1.14
22Rv1-KO5	0.04	0.10

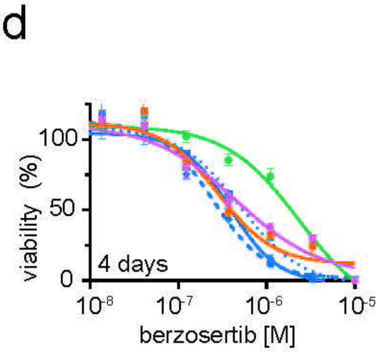

f

EC50 [nM]	ATR inhibitors berzosertib	elimusertib
LNCaP	598	77
C42B	400	41
189.4_CL	1905	160
22Rv1	399	91
22Rv1-KO2	540	57
22Rv1-KO5	295	34

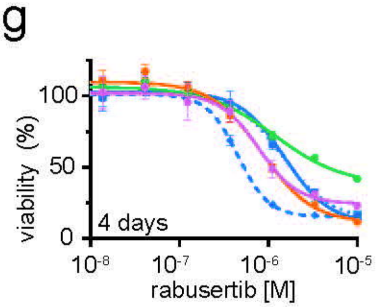

i

EC50 [μM]	CHEK1 inhibitors rabusertib	MK-8776
LNCaP	1.12	1.78
C42B	1.11	2.84
189.4_CL	4.55	>10
22Rv1	1.81	1.54
22Rv1-KO2	1.81	>10
22Rv1-KO5	0.53	0.48
