# Supplementary material for: Molecular consequences of acute versus chronic CDK12 loss in prostate carcinoma nominates distinct therapeutic strategies
Source: bioRxiv. 2024 Jul 19:2024.07.16.603734. Preprint. [Version 1] doi: 10.1101/2024.07.16.603734 (PMC11275783; doi:10.1101/2024.07.16.603734)
Supplement: Supplementary file 1 [file NIHPP2024.07.16.603734v1-supplement-1.pdf]

bioRxiv preprint doi: https://doi.org/10.1101/2024.07.16.603734; this version posted July 19, 2024. The copyright holder for this preprint (which was not certified by peer review) is the author/funder, who has granted bioRxiv a license to display the preprint in perpetuity. It is made available under aCC-BY 4.0 International license.

Supplemental Figure S1. CDK12 genomic alterations and tandem duplication phenotypes

(a) Tandem duplication (TD) size analysis confirms multiple modes of TD lengths (top) in whole exome (WES; orange) and whole genome sequencing (WGS; blue) data for 46 tumors with a TDP. Fitting mixtures of Gaussian distributions to each of 26 WES and 20 WGS samples provided estimates of Gaussian means. The distribution of these mean values across 26 WES cases and 20 WGS cases are shown in red dotted lines; samples may have multiple mean values, one for each estimated mixture. The mixture means are categorized into six possible TD Groups based on the length ranges (bottom). The proportion of each TD Group are shown for the four CRPC cohorts.

(b) The proportions of *CDK12*, *BRCA2*, and *TP53* genomic alteration status, including mono-allelic and bi-allelic losses and intact, for the 46 TDP cases compared across TD Groups.

Supplemental Figure S2. Gene expression alterations in *CDK12^BAL^* prostate cancers.

(a) GSEA of CDK12-mut up and down signatures reported in Wu et al., 2018 applied to mCRPC cohorts with versus without *CDK12^BAL^*. **(**Normalized enrichment scores (NES) shown in heatmap on plot, all enrichment false discovery rates (FDR) <0.0001).

(b-c) Overlap of differentially expressed genes up-regulated (**c**) or down-regulated **(d)** across mCRPC cohorts with versus without *CDK12^BAL^* (significance level shown on plot.)

(d-g) RNAseq based quantitation of AR pathway activity in mCRPC cohorts with versus without *CDK12^BAL^* (GSVA scores; Wilcoxon rank test p-values shown).

(h) Differential expression of genes contained within *CDK12^BAL^*-specific and recurrent fusions. * = Up-regulated or ^ down-regulated with p<0.05, FC>abs(2) in at least 1 cohort.

(i-l) RNAseq based transcript abundance levels of ATM in mCRPC cohorts with versus without *CDK12^BAL^* (Wilcoxon rank test p-values shown).

Supplemental Figure S3. APA usage in *CDK12^BAL^* prostate cancers.

(a-c) Association of increased/unchanged or decreased (p<0.05; FC<-2) transcript abundance levels in relation to gene size features in tumors without or with $\text{CDK12}^{\text{BAL}}$ in the HMF, SU2C-WC and UW mCRPC cohorts.

(d-f) APAlyzer analysis for up- and down-regulated APA usage using RNA-seq from mCRPC data sets comparing $\text{CDK12}^{\text{BAL}}$ cases vs CDK12(intact) controls: (c) TCGA-PRAD, (d) SU2C-I, and (e) HMF. RED = relative expression difference; each point is a different APA site.

bioRxiv preprint doi: https://doi.org/10.1101/2024.07.16.603734; this version posted July 19, 2024. The copyright holder for this preprint (which was not certified by peer review) is the author/funder, who has granted bioRxiv a license to display the preprint in perpetuity. It is made available under aCC-BY 4.0 International license.

(g) Transcript pile-up of reads mapping to exons demonstrating increased transcript reads corresponding to an IPA in the ATM gene in TCGA prostate (PRAD) and ovarian primary tumors with CDK12 alterations versus tumors with intact CDK12 and diminished transcripts mapping to the distal 3' exon.

Supplemental Figure S4. Effects of acute CDK12 inhibition.

(a) Treatment with ActD, Palbo, and SR4835 all lead to downregulation of longer transcripts. Distribution by transcript length (nucleotides/nt) of downregulated genes by RNA-seq (<-2 fold, FDR <0.05, 'n' depicted on plot) in LNCaP and LuCaP35_CL prostate cancer cell lines following six hours of exposure to vehicle (DMSO), CDK4/6 inhibitor palbociclib (Palbo, 10uM), broad RNA Pol-II inhibitor actinomycin D (ActD, 5ug/mL), or CDK12/13 inhibitor SR4835 (200nM) (n=3). Plots were made with ShinyGO 0.80 (76) and show significance (t-test) of downregulated vs upregulated or unchanged genes.

(b) CDK12 inhibition increases the number and ratio of upregulated APAs**.** Table with the number of APA sites down (DN), no change (NC), or up (UP) upon treatment vs vehicle and the ratio UP/DOWN, indicating any skew towards the IPA phenotype.

(c-d) Cell arrest and CDK12 inhibition both lead to HR pathway downregulation. **(c)** Selected KEGG pathway enrichment for DNA-repair related pathways focusing on changes from general G1/S arrest (palbociclib) vs CDK12 inhibition (SR4835). **(d)** Many SR4835 downregulated pathways overlap with cell cycle arrest. Venn diagram showing high overlap of KEGG pathways downregulated (NES <-1) with acute palbociclib or SR4835 treatment (6h).

(e-f) Effects of pharmacological CDK12/13 inhibition. Similar experiments as in Fig. 4e using LNCaP (**e**) and Skov3 (**f**) cells showing the effect on DNA repair and apoptotic proteins with SR4835 treatment with or without Z-VAD caspase inhibitor.

(g) LuCaP189.4 carries bi-allelic loss of function *CDK12* mutations. Diagram showing the two frameshift mutations carried by the LuCaP189.4 PDX upstream of the key functional kinase domain (yellow).

(h) LuCaP 189.4 does not express CDK12 protein. Immunohistochemical (IHC) staining (with amplification) for CDK12 on FFPE sections from LuCaP PDX tumors or cell spots from LuCaP189.4_CL and LuCaP189.4-CDK12 cell lines. Counterstained with hematoxylin.

(i) LuCaP189.4 shows hallmark TDP genomic pattern. Copy number plot from exome-seq of three LuCaP PDX lines (78CR, 174.1, and 189.4).

(j) *CDK12* knockout lines grow slower than parental lines. GFP tagged cells were grown for seven days and monitored by GFP imaging. Graphs show GFP confluence normalized to day 1 for each line with mean-/+stdev (n=5). Significance vs parental line was determined by two way ANOVA.

bioRxiv preprint doi: https://doi.org/10.1101/2024.07.16.603734; this version posted July 19, 2024. The copyright holder for this preprint (which was not certified by peer review) is the author/funder, who has granted bioRxiv a license to display the preprint in perpetuity. It is made available under aCC-BY 4.0 International license.

Supplemental Figure S5. Assessments of HR competency in cells with CDK12 loss.

(a) Stable CDK12(-) cells show fewer upregulated intronic APAs. Table with the number of APA sites down (DN), no change (NC), or up (UP) in isogenic paired models (CRISPR KO clones vs parental, or 189.4-CDK12 vs 189.4-vec). The ratio UP/DOWN indicates skew towards the IPA phenotype.

(b) Validation of Tet-shRNA lines. Western blot with lysates from Tet-shRNA lines treated four days -/+ 100ng/mL doxycycline.

(c) LuCaP189.4_CL cells are RAD51 competent. Irradiation and immunostaining (same as in Fig 5f). Cells were exposed to 6Gy IR and fixed at 3h. Immunofluorescence staining was performed for γH2A.X and RAD51 and images were acquired by confocal microscopy. Left: representative images (white: DAPI, green: γH2A.X, purple: RAD51). Right: quantification of images (~200-500 cells analyzed per treatment). Line is at mean and significance was determined by unpaired t-test (Mann-Whitney).

(d-e) *CDK12* knockdown does not prevent RAD51 foci. Additional immunostaining (same as in Fig 5f) using LNCaP (d) and Skov3 (e) with Tet-shCDK12 or Tet-shBRCA2. Cells were treated four days -/+ dox. Graphs show mean with significance determined by one-way ANOVA (Kruskal-Wallis).

Supplemental Figure S6. Drug sensitivities in prostate cancers with CDK12 loss.

(a-b) CDK12 loss does not confer HRd-expected platinum or PARPi sensitivity. Dose response curves for prostate cancer (a) or ovarian cancer (b) cell lines treated 8 days with carboplatin (n=3 for prostate lines, n=4 for ovarian lines). EC50 values are shown on the legend.

(c) Ovarian cancer cells (n=4) were treated 12 days with olaparib.

(d) Prostate cancer lines and UWB1.289 (n=3) were treated 12 days with olaparib.

(e) Prostate cancer lines and UWB1.289 (n=3) were treated 8 days with rucaparib.

(f-g) LuCaP189.4 organoids do not show obvious PARPi sensitivity. LuCaP PDX tumors were dissociated into organoids and treated (at passage 3) with olaparib **(f)** or rucaparib **(g)** for 14 days. Plots show mean-/+stdev (n=4).

(h-j) CDK13 sgRNA is detrimental in 22Rv1 lacking *CDK12*. Experiment as in Fig 6f. GFP tagged 22Rv1 or 22Rv1-CDK12-KO5 were transduced with sgRNAs and monitored by imaging. Example images are in (h). Plots show growth rates for 22Rv1 (**i**) and 22Rv1-CDK12-KO5 (**j**) by confluence (%GFP--/+stdev, n=5) with significance vs sgAAVS1 determined by two-way ANOVA.

(k-m) LuCaP189.4_CL shows sensitivity to CDK13 inhibition. Dose response curves from four prostate cancer lines, including CDK12^{BAL} LuCaP189.4_CL, treated six days with the CDK12/13 inhibitors SR4835 (k) or THZ531 (l) and a table (m) with the calculated EC50 values.

(n) APAlyzer analysis from SR4835 treated PDX tumors. Three tumors of each group from Fig. 6j plus

bioRxiv preprint doi: https://doi.org/10.1101/2024.07.16.603734; this version posted July 19, 2024. The copyright holder for this preprint (which was not certified by peer review) is the author/funder, who has granted bioRxiv a license to display the preprint in perpetuity. It is made available under aCC-BY 4.0 International license.

3 day treated LuCaP189.4 were analyzed by RNA-seq. The table shows the number of intronic APA sites down (DN), no change (NC), or up (UP) in treated vs vehicle comparisons. The ratio UP/DOWN indicates skew towards the IPA phenotype.

Supplemental Figure S7. Effects of WEE1, ATR and CHEK1 inhibitors toward prostate cancers with CDK12 loss.

(a-c) One CDK12-KO prostate line, 22Rv1-CDK12-KO5, shows increased sensitivity to WEE1 inhibition. Prostate cancer lines were treated with WEE1 inhibitors adavosertib/MK-1775 (a) and PD0166285 (b) for four days. Dose response curves show relative viability vs drug concentration. Plots show mean-/+stdev (n=4). Legend and EC50 values (µM) are shown in (c).

(d-f) CDK12-KO prostate lines do not show sensitivity to ATR inhibitors. Prostate cancer lines were treated with ATR inhibitors berzosertib/VX-970 (d) and elimusertib/BAY-1895344 (e) for four days. Dose response curves show relative viability vs drug concentration. Plots show mean-/+stdev (n=4). Legend and EC50 values (nM) are shown in (f).

(**g-i**) One CDK12-KO prostate line, 22Rv1-CDK12-KO5, shows increased sensitivity to CHEK1 inhibition. Prostate cancer lines were treated with CHEK1 inhibitors rabusertib (g) and MK-8776 (h) for four days. Dose response curves show relative viability vs drug concentration. Plots show mean-/+stdev (n=4). Legend and EC50 values (µM) are shown in (i).

FIGURE S1

a

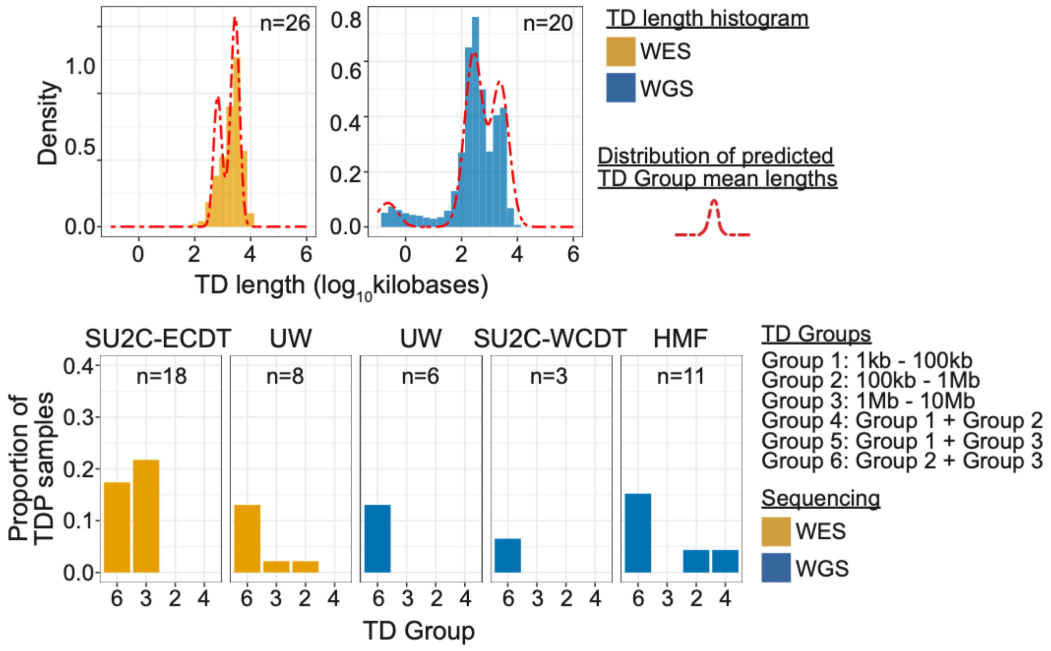
n=26
n=20
Density
TD length (log10kilobases)
TD length histogram
WES
WGS
Distribution of predicted
TD Group mean lengths
SU2C-ECDT
UW
UW
SU2C-WCDT
HMF
n=18
n=8
n=6
n=3
n=11
Proportion of TDP samples
TD Group
TD Groups
Group 1: 1kb - 100kb
Group 2: 100kb - 1Mb
Group 3: 1Mb - 10Mb
Group 4: Group 1 + Group 2
Group 5: Group 1 + Group 3
Group 6: Group 2 + Group 3
Sequencing
WES
WGS

b

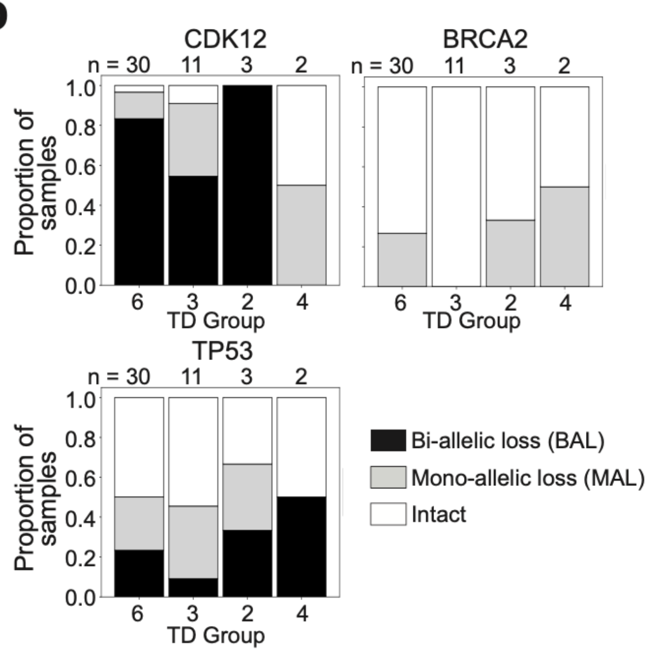
CDK12
BRCA2
TP53
n = 30
11
3
2
Proportion of samples
TD Group
Bi-allelic loss (BAL)
Mono-allelic loss (MAL)
Intact

FIGURE S2

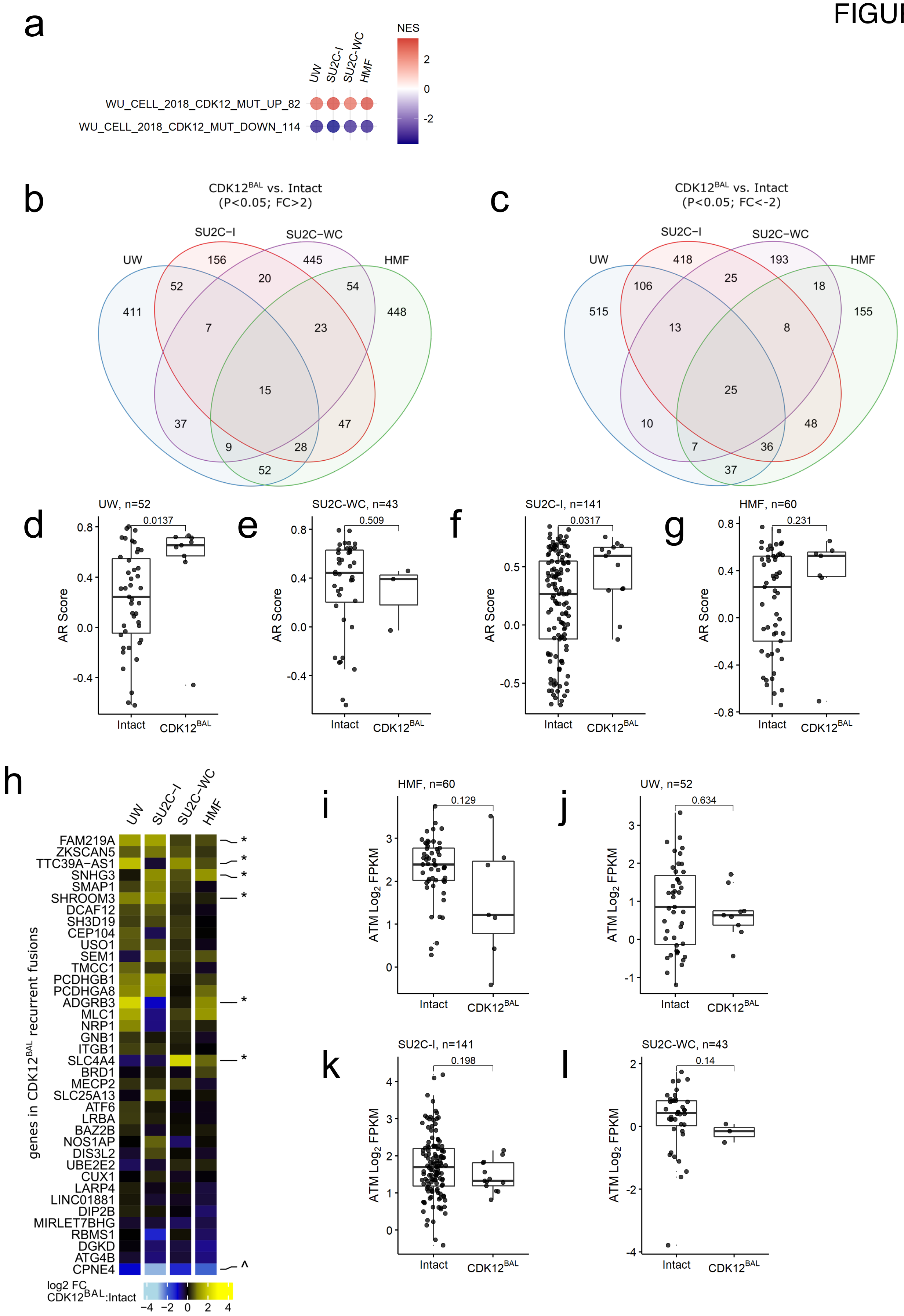
a
UW
SU2C-I
SU2C-WC
HMF
NES
2
0
-2
WU_CELL_2018_CDK12_MUT_UP_82
WU_CELL_2018_CDK12_MUT_DOWN_114
b
CDK12BAL vs. Intact
(P<0.05; FC>2)
UW
SU2C−I
SU2C−WC
HMF
411
156
445
448
52
20
54
7
23
15
37
47
9
28
52
c
CDK12BAL vs. Intact
(P<0.05; FC<-2)
UW
SU2C−I
SU2C−WC
HMF
515
418
193
155
106
25
18
13
8
25
10
48
7
36
37
d
UW, n=52
0.0137
AR Score
Intact
CDK12BAL
e
SU2C-WC, n=43
0.509
AR Score
Intact
CDK12BAL
f
SU2C-I, n=141
0.0317
AR Score
Intact
CDK12BAL
g
HMF, n=60
0.231
AR Score
Intact
CDK12BAL
h
UW
SU2C−I
SU2C−WC
HMF
genes in CDK12BAL recurrent fusions
FAM219A
ZKSCAN5
TTC39A−AS1
SNHG3
SMAP1
SHROOM3
DCAF12
SH3D19
CEP104
USO1
SEM1
TMCC1
PCDHGB1
PCDHGA8
ADGRB3
MLC1
NRP1
GNB1
ITGB1
SLC4A4
BRD1
MECP2
SLC25A13
ATF6
LRBA
BAZ2B
NOS1AP
DIS3L2
UBE2E2
CUX1
LARP4
LINC01881
DIP2B
MIRLET7BHG
RBMS1
DGKD
ATG4B
CPNE4
log2 FC
CDK12BAL:Intact
−4 −2 0 2 4
i
HMF, n=60
0.129
ATM Log2 FPKM
Intact
CDK12BAL
j
UW, n=52
0.634
ATM Log2 FPKM
Intact
CDK12BAL
k
SU2C-I, n=141
0.198
ATM Log2 FPKM
Intact
CDK12BAL
l
SU2C-WC, n=43
0.14
ATM Log2 FPKM
Intact
CDK12BAL

FIGURE S3

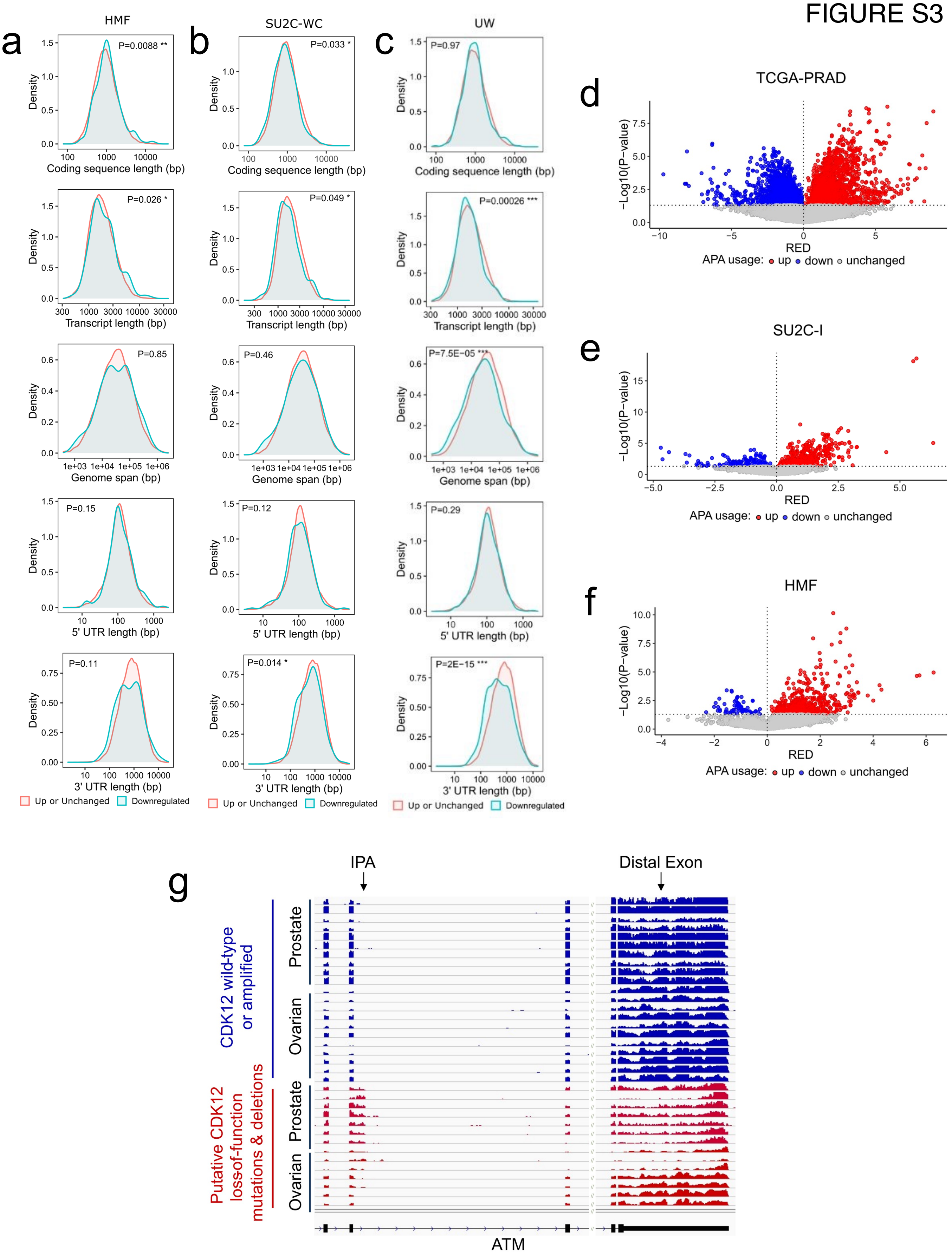

a
b
c
d
e
f
g
HMF
SU2C-WC
UW
P=0.0088 **
P=0.033 *
P=0.97
Coding sequence length (bp)
P=0.026 *
P=0.049 *
P=0.00026 ***
Transcript length (bp)
P=0.85
P=0.46
P=7.5E-05 ***
Genome span (bp)
P=0.15
P=0.12
P=0.29
5' UTR length (bp)
P=0.11
P=0.014 *
P=2E-15 ***
3' UTR length (bp)
Density
Up or Unchanged
Downregulated
TCGA-PRAD
SU2C-I
HMF
−Log10(P−value)
RED
APA usage: up down unchanged
IPA
Distal Exon
Prostate
Ovarian
CDK12 wild-type or amplified
Putative CDK12 loss-of-function mutations & deletions
ATM

FIGURE S4

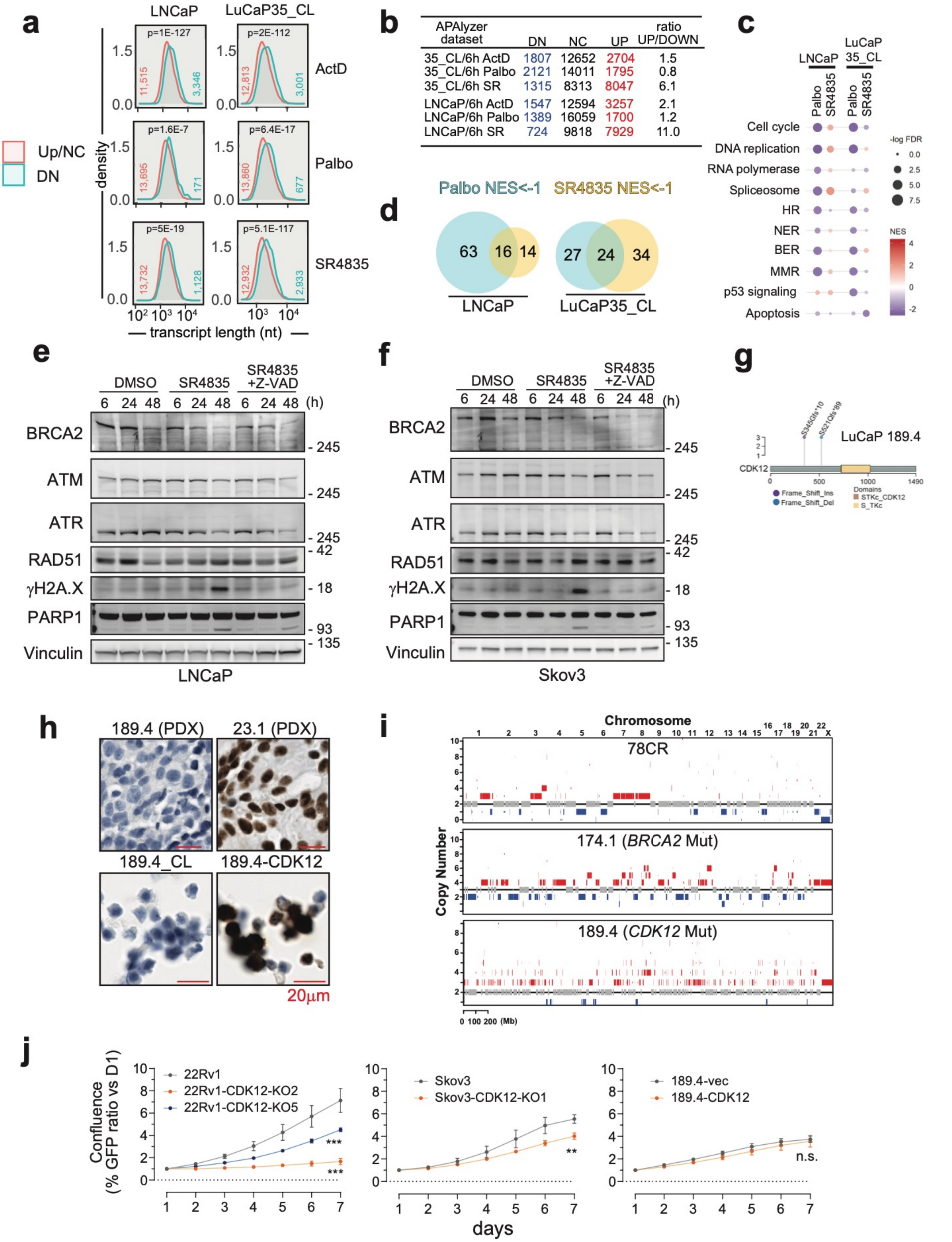
a
LNCaP
LuCaP35_CL
p=1E-127
p=2E-112
ActD
11,515
3,346
12,813
3,001
Up/NC
DN
density
p=1.6E-7
p=6.4E-17
Palbo
13,695
171
13,860
677
p=5E-19
p=5.1E-117
SR4835
13,732
1,128
12,932
2,933
transcript length (nt)
b
APAlyzer dataset | DN | NC | UP | ratio UP/DOWN
35_CL/6h ActD | 1807 | 12652 | 2704 | 1.5
35_CL/6h Palbo | 2121 | 14011 | 1795 | 0.8
35_CL/6h SR | 1315 | 8313 | 8047 | 6.1
LNCaP/6h ActD | 1547 | 12594 | 3257 | 2.1
LNCaP/6h Palbo | 1389 | 16059 | 1700 | 1.2
LNCaP/6h SR | 724 | 9818 | 7929 | 11.0
c
LNCaP
LuCaP 35_CL
Palbo
SR4835
Cell cycle
DNA replication
RNA polymerase
Spliceosome
HR
NER
BER
MMR
p53 signaling
Apoptosis
-log FDR
NES
d
Palbo NES<-1
SR4835 NES<-1
63
16
14
27
24
34
LNCaP
LuCaP35_CL
e
DMSO
SR4835
SR4835 +Z-VAD
6 24 48 6 24 48 6 24 48 (h)
BRCA2
ATM
ATR
RAD51
γH2A.X
PARP1
Vinculin
- 245
- 245
- 245
- 42
- 18
- 93
- 135
LNCaP
f
DMSO
SR4835
SR4835 +Z-VAD
6 24 48 6 24 48 6 24 48 (h)
BRCA2
ATM
ATR
RAD51
γH2A.X
PARP1
Vinculin
Skov3
g
LuCaP 189.4
CDK12
Frame_Shift_Ins
Frame_Shift_Del
Domains
STKc_CDK12
S_TKc
h
189.4 (PDX)
23.1 (PDX)
189.4_CL
189.4-CDK12
20μm
i
Chromosome
78CR
174.1 (BRCA2 Mut)
189.4 (CDK12 Mut)
Copy Number
0 100 200 (Mb)
j
Confluence (% GFP ratio vs D1)
22Rv1
22Rv1-CDK12-KO2
22Rv1-CDK12-KO5
Skov3
Skov3-CDK12-KO1
189.4-vec
189.4-CDK12
***
***
**
n.s.
days

FIGURE S5

a

APAlyzer dataset	DN	NC	UP	ratio UP/DOWN
22Rv1-KO2	2651	14322	2161	0.8
22Rv1-KO5	1926	14561	2350	1.2
Skov3-KO1	1229	12259	3347	2.7
189.4-CDK12	1791	16031	804	0.4

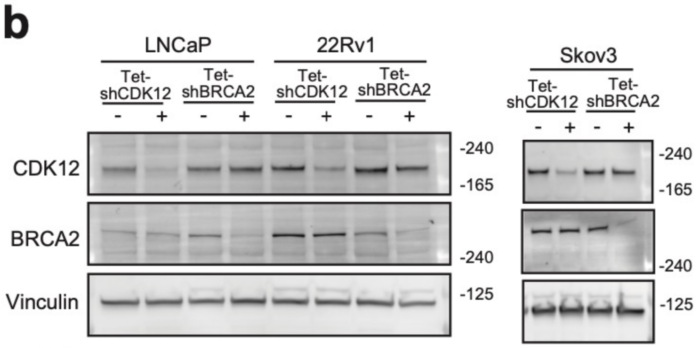

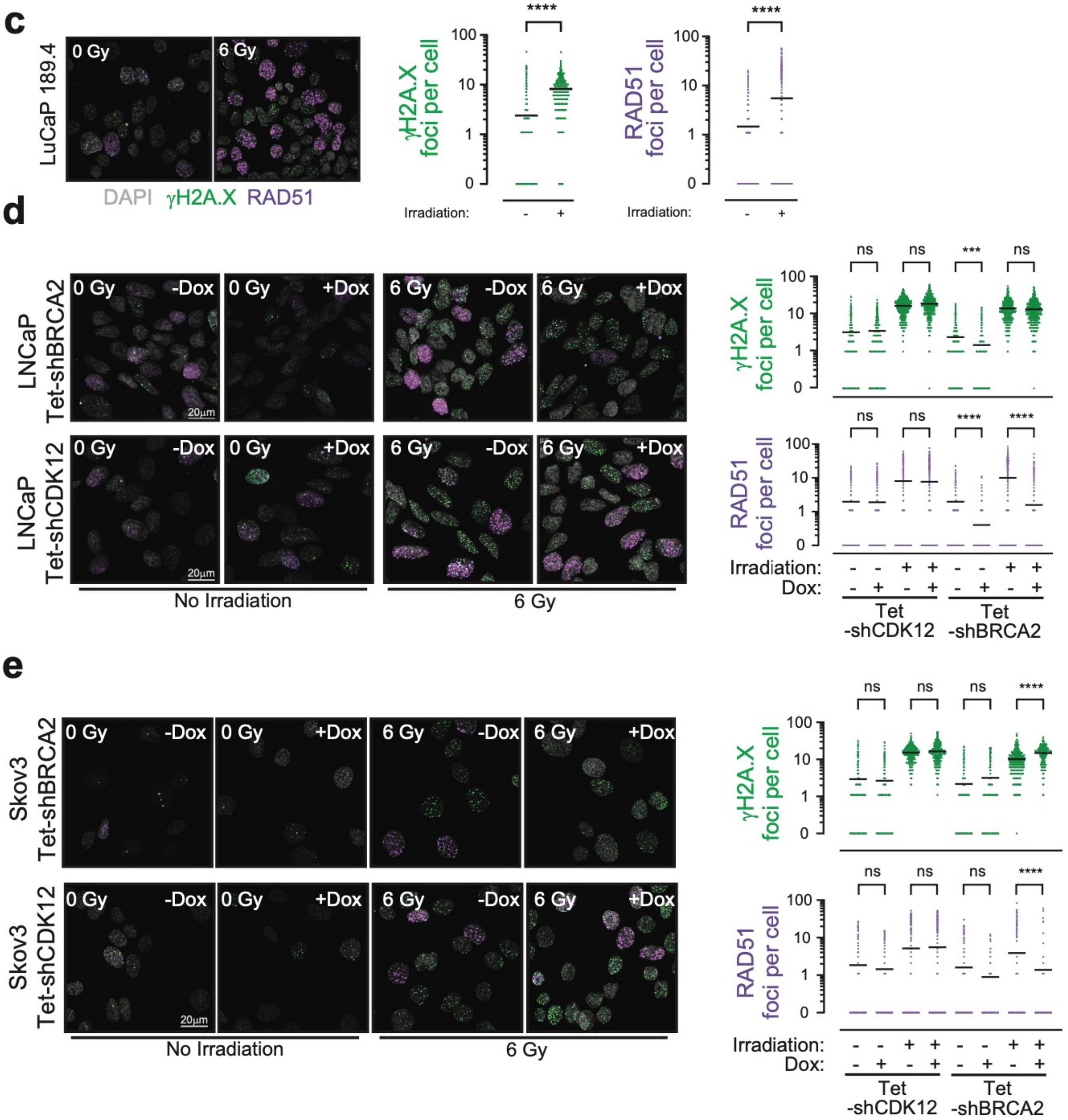

FIGURE S6

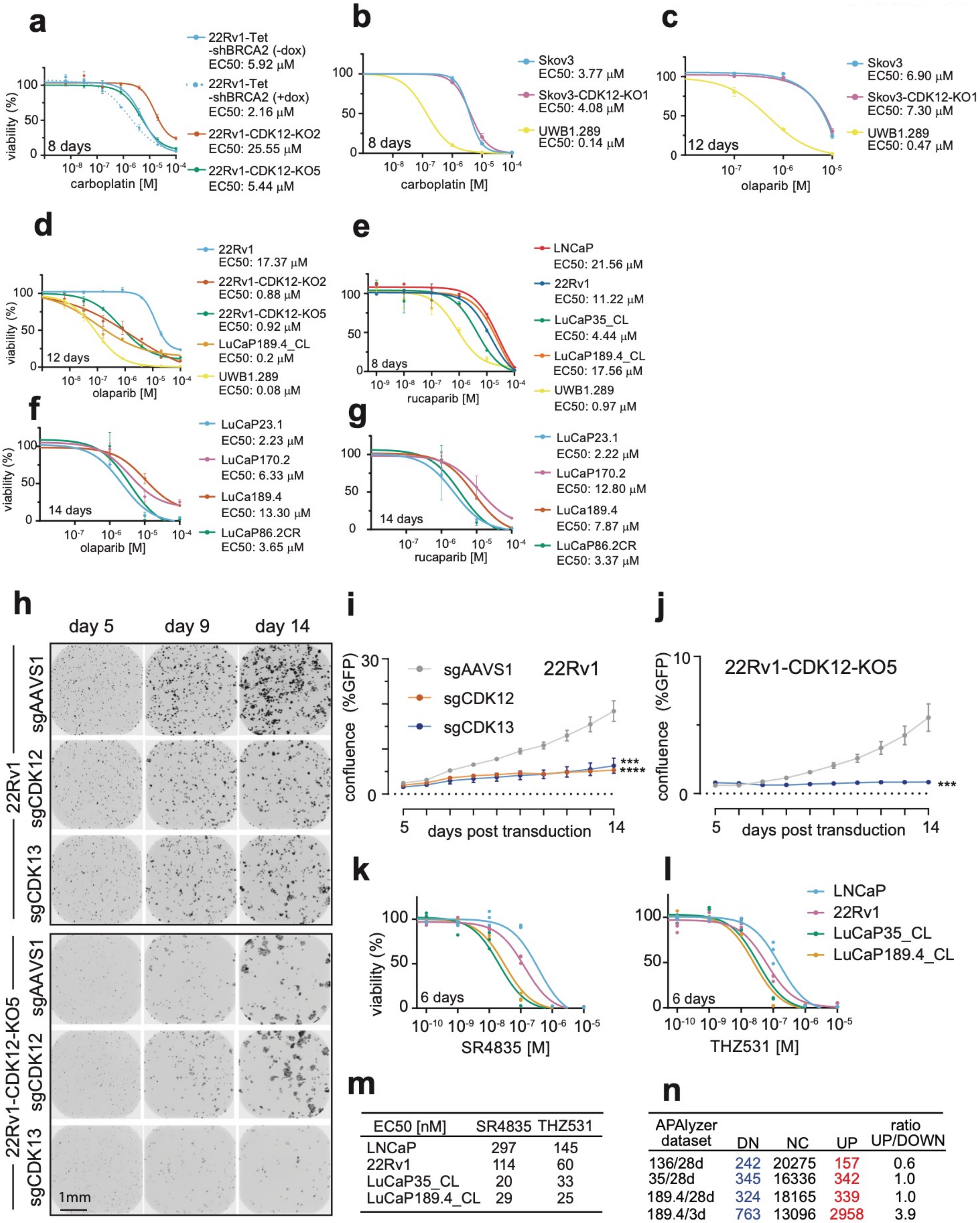

m

EC50 [nM]	SR4835	THZ531
LNCaP	297	145
22Rv1	114	60
LuCaP35_CL	20	33
LuCaP189.4_CL	29	25

n

APAlyzer dataset	DN	NC	UP	ratio UP/DOWN
136/28d	242	20275	157	0.6
35/28d	345	16336	342	1.0
189.4/28d	324	18165	339	1.0
189.4/3d	763	13096	2958	3.9

FIGURE S7

a **b**

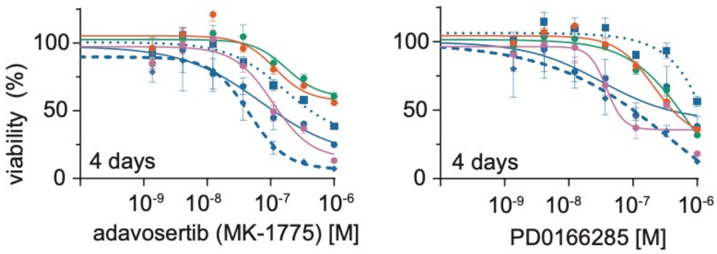

c

EC50 [μM]	WEE1 inhibitors adavosertib	PD0166285
LNCaP	0.13	0.06
189.4-vec	>1	0.41
189.4-CDK12	>1	0.53
22Rv1	0.11	0.37
22Rv1-KO2	0.43	1.14
22Rv1-KO5	0.04	0.10

d **e**

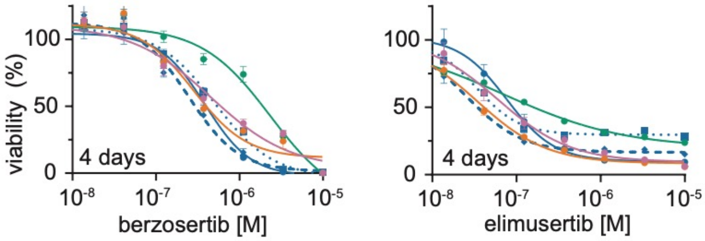

f

EC50 [nM]	ATR inhibitors berzosertib	elimusertib
LNCaP	598	77
C42B	400	41
189.4_CL	1905	160
22Rv1	399	91
22Rv1-KO2	540	57
22Rv1-KO5	295	34

g **h**

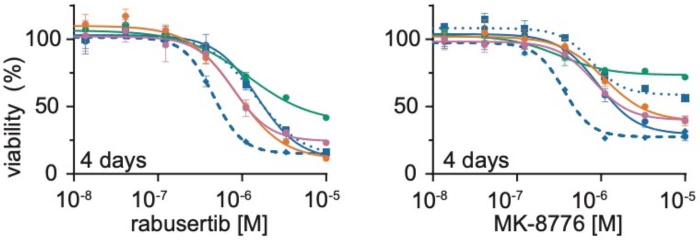

i

EC50 [μM]	CHEK1 inhibitors rabusertib	MK-8776
LNCaP	1.12	1.78
C42B	1.11	2.84
189.4_CL	4.55	>10
22Rv1	1.81	1.54
22Rv1-KO2	1.81	>10
22Rv1-KO5	0.53	0.48
